# Food safety governance in China: From supervision to coregulation

**DOI:** 10.1002/fsn3.1281

**Published:** 2019-11-20

**Authors:** Zhe Liu, Anthony N. Mutukumira, Hongjun Chen

**Affiliations:** ^1^ School of Management Henan University of Technology Zhengzhou China; ^2^ School of Food and Nutrition Massey University Auckland New Zealand

**Keywords:** coregulation, food safety, governance, regulatory system

## Abstract

The food control and regulatory system in China is beset by several challenges. While firms have to reduce their costs in pursuit of benefits, customers are increasingly focusing on safety and quality of food products. Although the Chinese government has developed more stringent regulatory measures, food safety incidents still occur, including abuse of food additives, adulterated products as well as contamination by pathogenic microorganisms, pesticides, veterinary drug residues, and heavy metals, and use of substandard materials. A national food safety strategy has been proposed to assure food safety from “farm to table.” This paper begins with the analysis of current food regulatory systems and then discusses cogovernance of food safety management in China. We explore the practice in the city of Shenzhen where government intervention has strengthened food control, thereby creating an opportunity to form a coregulatory system. The review highlights that the current food safety regulatory system of multi‐agency structure can inevitably lead to insufficient incentives for business entities. Due to asymmetric information, lack of regulatory resources, and consumer advocacy, coregulation has been developed and is increasingly being promoted as an important instrument of food regulation.

## Graphical abstract

In this manuscript, we divide the development of Chinese food safety regulation into five stages, including centralized management (1949–1979), multisector management (1979–1995), matrix management (1995–2009), process management (2009–2015), and integrated management (2015 to date), and show that there are still many challenges from stakeholders, technology, and resources. Due to asymmetric information, lack of regulatory resources, and consumer advocacy, coregulation has been developed and is increasingly being promoted as an important instrument of regulation. The paper presents a conceptual framework of enforcement of food safety regulation for use in shifting toward coregulation from traditional approaches, based on the case study of Shenzhen.

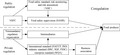

## 1 INTRODUCTION

Despite the growing recognition of food safety as a public health priority and as an essential requirement for food trade, food safety incidents still occur occasionally. Several food safety incidents have been exposed annually by the China Central Television during a consumer program to mark World Consumer Rights Day. One serious food safety incident was recently reported which involved unsanitary conditions at a factory which makes spicy gluten strips. The snack strips are popular among teenagers and omnipresent at food stalls near schools because they are inexpensive. In another reported case, 36 students were hospitalized for diarrhea or stomach pains after consuming contaminated food in a local “model student canteen.” The food was suspected to be contaminated by microorganisms due to visible growth of molds (China Daily, 2019). Those incidents were not caused by lack of or limited technological resources but more by poor understanding of food safety and its implications, or food safety culture, which often results in a downgrading of food safety to “low priority” in the food producers’ agenda (Powell, Jacob, Chapman, 2011; Yiannas, 2009). Food safety problems are different at different stages of economic development. Therefore, this paper discusses the changes of food safety governance in China from the historical perspective.

When developing food safety policies, a range of factors should be considered including international regulations and accepted approaches, private sector, consumer interests and requirements, political will, and socioeconomic issues in addition to science and risk assessments. Considering and balancing these factors are country‐specific. For instance, in countries with developed economies, governments have compliance‐based enforcement systems with emphasis on preventing hazards from occurring, compared to deterrent‐based strategies, based on tiered inspection regimes (Garcia Martinez, Fearne, Caswell, & Henson, 2007; Fulponi, 2006; Lee & Marsden, 2009; Loader & Hobbs, 1999; Rouvière & Caswell, 2012; Rouvière, Soubeyran, & Bignebat, 2010).

Since the 1990s, food safety regulations have evolved worldwide and food producers have frequently been given more responsibility to monitor the safety of their products (Codron, Fares, & Rouvière, 2007; Henson & Caswell, 1999; Henson & Hooker, 2001; Loader & Hobbs, 1999; Segerson, 1999). This led to the introduction of the Hazard Analysis Critical Control Point (HACCP) system in several food industries worldwide. As a result of its success in countries with developed economies, HACCP is increasingly being implemented in countries with less developed economies that export food products into industrialized markets (Merican, 1996). The growing use of HACCP as a food safety management system in international trade led the Codex Alimentarius to develop guidelines for HACCP in 1993 and incorporate it into food hygiene codes starting in 1995 (Whitehead & Orriss, 1995). Despite the widespread acceptance of the application of HACCP among regulatory and international agencies, there are several controversies surrounding enforcement of the food safety system (Antle, 1998; Hathaway, 1995; Hathaway & Cook, 1997; Smith‐De Waal, 1996). Considering the scarcity of public sector resources, impact of the system on competitiveness, and the scale of the workload, there are growing interests in coregulation, with public and private sectors working together to deliver safe food at low regulatory cost (Garcia Martinez, Fearne, Caswell, & Henson, 2007). There should be a combination of responsibility from all levels of stakeholders in the food retail sector in order to improve food safety and prevent food safety breaches (Boatemaa et al., 2019). Increasing number of countries uses new coregulation schemes focusing on a specific type of coregulation where regulations are developed by public authorities and then implemented by the coordinated actions of public authorities and food operators or “enforced self‐regulation” (Guo, Bai, & Gong, 2019; Rouvière & Caswell, 2012).

From a theoretical perspective, the existence of market failures is the central rationale for regulatory intervention in the provision of food safety. The market failure is attributed to the existence of asymmetric information about food safety attributes between producers and consumers or imperfect, symmetric information for both consumers and producers (Antle, 1996). The level of public intervention may range from being inactive, thereby leaving the market to find the requisite solution to direct regulation (Better Regulation Task Force, 2003). In‐between, there is a wide range of options including industry self‐regulation, government provision of information, education campaigns, and labeling requirements (Garcia Martinez et al., 2007). The consequent problem is the relationships between regulators and the regulated and the scope for regulatory capture which is the pursuit of the regulated business’ interests rather than those of the public at large (Fearne et al., 2004). Coregulation aims to combine the advantages of the predictability and binding nature of legislation with the flexibility of self‐regulatory approaches. It would therefore serve as a possible model for food safety governance, mixing self‐regulation and legislative action working together in a manner that mutually reinforces one another (Dordeck‐Jung, Oude Vrielink, Gosselt, Hoof, & Jong, 2010; Eijlander, 2005; Garcia Martinez et al., 2007; Goldsmith & Wu, 2006; Gunningham & Grabosky, 1998).

Food safety regulation receives particular attention in China due to complex, highly publicized food safety incidents (Lu & Wu, 2014). In this paper, we focus on the situation in China where the food control system is undergoing a shift from supervision to social coregulation. The food control system in China was not well organized before 2009. In 2008, about 294,000 infants were diagnosed with urinary calculus, and more than 50,000 were hospitalized, while six infants died (MOH, 2008). The first Food Safety Law was released by the Chinese government in February 2009 and implemented in June of the same year. The Food Safety Law has been delayed for a long time, due to the development of various versions of the law by several major regulatory departments, resulting in lack of consensus. The concept of “Healthy China” was proposed at the 19th National Congress of the Communist Party of China in 2017. In the proposal, China is expected to improve the national health policy and the system for medicine supply, thereby promoting healthy and positive lifestyles, and initiating a food safety strategy to ensure that people have confidence in the food they eat (Xi, 2017).

The proposed classical regulatory system consists of three levels: rules, supervision, and execution. Laws and regulations related to food safety in China are often drafted by specific law enforcement departments, such as Food Hygiene Law, Product Quality Act, Consumer Protection Act, and Agricultural Product Quality Act. The supervision of food safety is mainly dependent on law enforcement departments. Therefore, food safety regulatory authorities can reflect the basic vision of China's food safety regulation. As the highest regulatory authority in China, The Food Safety Committee of the State Council suggested that the food safety management must be further strengthened with strict standards, strict supervision, strict punishment, and strict accountability system to ensure the “tongue‐in‐tongue safety” of the people (SC, 2019). Shenzhen won 10% of the total votes of 67 pilot cities in China, ranking first, during the “Thumbs up for Your City” campaign of the Food Safety Committee. The successful experiences of Shenzhen showed that once the coregulation system was formed, it needs to be supported immediately by the regulatory authorities with finance, propaganda, and legitimacy. This paper is presented in five parts: The next section discusses the history of food safety governance in China, and the subsequent sections focus on challenges and weaknesses of the current food control and regulatory system up to date, and strengths and opportunities of coregulation practice in the city of Shenzhen. The paper concludes with some thoughts on the role of coregulation in food safety and the need for further research.

## 2 HISTORY OF FOOD SAFETY GOVERNANCE IN CHINA

At the beginning of China's economic reform, more than 82% of the country's population lived in rural areas and people consumed mostly raw, homemade, and less processed foods. In the last three decades, as industrialization and urbanization accelerates, the proportion of the rural population has reduced to about 40% (Chinese Statistical Yearbook, 2018). Due to the successful cultivation of hybrid wheat and hybrid rice varieties and the application of fertilizer, China has been self‐sufficient of grain since the 1990s. Modernization of China's food industry has resulted in increasing animal food production and growing capacity of food processing. Chinese consume much more processed and packaged foods, and often away from home (Liu, Wahl, Seale, & Bai, 2015; Waldron, Brown, & Longworth, 2010; Zhai et al., 2014; Zhang, Wu, Yao, Bai, & Xiong, 2014; Zhou, Zhang, & Xu, 2012).

In order to maintain efficient agricultural production, the Chinese government has invested in the production technology of rice, wheat, corn, cotton, soybean, pig, cattle, and sheep, including transgenic technology research. In recent years, serious environmental pollution has brought crisis to agricultural water (Lu et al., 2015). In addition, a large number of pesticides are used in China's cultivated land. China has been a country with the largest amount of pesticide production in the world. The rapid transformation of China's food industry with massive food processing could increase the possibility for opportunistic behavior in food malpractices, resulting in the emergence of food fraud or economically motivated adulteration in the country (Hong & Wu, 2017; Zhang & Xue, 2016).

In this section, the development of the Chinese food safety regulation was divided into five stages (Table 1), comprising the centralized management (1949–1979), multisector management (1979–1995), matrix management (1995–2009), process management (2009–2015), and integrated management (2015 to date). While the Chinese government has developed more stringent regulatory measures and established the State Administration for Market Regulation, the intermediary management of the current regulatory system may be the Achilles' heel, as shown in section 3. Due to asymmetric information, lack of regulatory resources, and consumer advocacy, coregulation has been developed and is increasingly being promoted as an important instrument of regulation, as discussed in section 4.

**Table 1:** Periods of Chinese food safety regulation

Food hygiene management			Food safety governance	
Ⅰ	Ⅱ	Ⅲ	Ⅳ	Ⅴ
1949–1979	1979–1995	1995–2009	2009–2015	2015‐
Centralized Management	Multisector Management	Matrix Management	Process Management	Integrated Management
Ministry of Health; Departments of five ministries involved	Departments of agriculture, forestry, animal husbandry, aquaculture, grain, supply and marketing, business, light industry, and trade.	Institutions of the Ministry of Health at all levels over the country; Departments of local government in the jurisdiction.	Food Safety Committee for overall guidance; Relevant Ministries for process management	Food Safety Committee for overall guidance; CFDA (SAMR, 2018) for safety supervision; NHFPC (NHC, 2018) for risk monitoring, risk assessment, and safety standard.
Regulations on the Management of Food Hygiene (Trial Implementation) 1964	Food Hygiene Law (Trial Implementation) 1982	Food Hygiene Law 1996	Food Safety Law 2009	Food Safety Law (Amended) 2015

### 2.1 Period One: 1949–1979

In the 1950s, like most regions of the world, the main food concern of the Chinese government was on food supply. The concern for food safety mainly came from diseases and foodborne illnesses caused by health problems (Jen & Chen, 2017). In 1964, the State Council promulgated the trial regulations on the implementation of food hygiene management. Although the regulation still focuses on preventing foodborne illnesses and intestinal infectious diseases, it has reflected the transition from single process to comprehensive management. The regulation includes food management departments of five ministries and commissions: the Ministry of Health, Ministry of Commerce, Ministry of Light Industry, the Central Administration of Industry and Commerce, and China Federation of Supply and Marketing Cooperatives.

The Ministry of Health was in charge of food safety during this period. However, food safety, from the perspective of health, was mainly undertaken by health supervision system. The health supervision system had undergone through complicated circuitous reform from health and epidemic prevention agencies, including law enforcement, scientific research, and technology services, to independent institutions of health supervision.

### 2.2 Period Two: 1979–1995

In 1979, multisector management situation emerged. The State Council issued the regulations on food hygiene management, and the emphasis of food hygiene management transferred from prevention of intestinal infectious diseases to prevention of foodborne diseases. The 7th article pointed out that all departments of agriculture, forestry, animal husbandry, aquaculture, grain, supply and marketing, business, light industry, and trade should strengthen the purchase and inspection work on grain, oil, meat, eggs, aquatic products, vegetables, fruits, tea, and other food ingredients, and need to strictly prevent industrial waste, radioactive substances, pesticide pollution, and the spread of animal diseases (RMFH, 1979).

In 1982, the People's Republic of China Food Hygiene Law (Trial Implementation) was promulgated and implemented, and food hygiene supervision system was clearly defined. It also stipulated that the food production and operation enterprises are responsible for the food hygiene work (FHL, 1982). The administrative department of industry and commerce administered urban and rural affairs of food hygiene management and food hygiene inspection work. The department of agriculture and animal husbandry and fishery assumed the management of veterinary hygiene inspection of livestock and poultry. The health and epidemic prevention station or the institute of food hygiene supervision and inspection of the health administrative department was responsible for food hygiene supervision within the jurisdiction. The imported food products were inspected by the state food hygiene supervision and inspection agency. The exported food was supervised and inspected by the national import and export commodity inspection departments.

### 2.3 Period Three: 1995–2009

In October 1995, the Food Hygiene Law of the People's Republic of China defined the operation of food hygiene supervision and stressed that food management departments at various levels shall strengthen the administration of food hygiene. The Ministry of Health administered the supervision and management of national food hygiene, while the relevant departments of local government were responsible for food hygiene management in the jurisdiction (FHL, 1995). The duties of the administrative departments for Industry and Commerce and the duties of the imported and exported inspection departments remained unchanged.

In 1998, the State Bureau of Quality and Technical Supervision was established and, according to the Products Quality Law, began to administer the food quality and safety supervision. In 1999, according to the Notice of Office of the State Council on the Allocation of Functions, Internal Structure, and Staffing Requirements of the National Entry‐exit Inspection and Quarantine Bureau, the functions of examination, approval, release of national standard of food hygiene, and the guidance and supervision of the quality of pesticide were undertaken by the Bureau of Quality and Technical Supervision (PQL, 2000). Meanwhile, the State Council decided to merge the former State Commodity Inspection Bureau, State Animal and Plant Quarantine Bureau, and the State Health Quarantine Bureau into the National Entry‐exit Inspection and Quarantine Bureau. The functions of legislation are allocated to health, agriculture, and other departments, and the functions of quality inspection have been integrated.

To strengthen market supervision, the State Council merged the former National Entry‐exit Inspection and Quarantine Bureau and the State Bureau of Quality and Technical Supervision into the General Administration of Quality Supervision, Inspection and Quarantine in April 2001.

At the beginning of 2002, Chinese Center for Disease Control and Prevention (CDC) and Health Supervision Center were established by the Ministry of Health, while the former was required to provide technical support and complete disease control and public health service, and the latter was expected to implement the functions of public health administration.

In 2003, the State Food and Drug Administration (SFDA) was set up on the basis of the State Drug Administration. The establishment of SFDA marked the development of the national food safety regulatory agency as an independent law executor.

In 2004, the Decision of the State Council on Further Strengthening the Work of Food Safety and Information on Food Safety Supervision Departments to Further Clarifying the Division of Responsibilities of Relevant Issues presented food safety supervision was co‐executed by the AQSIQ (Administration of Quality Supervision, Inspection and Quarantine), the State Administration for Industry and Commerce, the Ministry of Health, Ministry of Agriculture, the SFDA (State Food and Drug Administration), and the National Standardization Committee. The principle of division supervision was intended to improve the food safety regulatory functions and to clarify the responsibilities: The agriculture departments assumed responsibility for supervision of the production of primary agricultural products; quality inspection departments were responsible for food production and processing; the quality inspection departments were responsible for food production, which were previously under the health department; departments of industry and commerce were responsible for the supervision of the consumption sections, such as restaurants and canteens; and departments of state food and drug regulatory authorities were responsible for the comprehensive supervision of food safety, organization, coordination, investigation, and punishment for serious accidents. According to the principle of consistency of rights and responsibilities, food safety regulatory responsibility and accountability system was established. In addition, to strengthen the management and comprehensive utilization of food information, the interdepartmental information communication platform was used to achieve interoperability and resource sharing. In July 2007, the State Council emphasized the above functions and principles of the Special Rules of the State Council on Strengthening the Supervision and Management of the Safety of Food and Other Products (SC, 2007).

### 2.4 Period Four: 2009–2015

In June 2009, the Food Safety Law was implemented with emphasis on food safety regulation transferred from food hygiene management to food safety governance. In February 2010, The State Council established the Food Safety Committee, which commenced administration by analyzing the situation of food safety, deployment of the overall guidance work, presenting major policy measures, and supervising food law implementation. As the highest coordinating body, the Food Safety Committee comprised of three vice premiers and 15 ministers.

The Ministry of Health was responsible for food safety risk monitoring and assessment, food safety standards, food safety information disclosure, food inspection agency qualification and inspection norms, investigation, and handling of major food safety accidents, such as clenbuterol incident of Shuanghui, gutter oil scandal, plasticizer contamination, and cadmium‐tainted rice. The function of food safety supervision was separated from the public health administrative departments at all levels.

The Ministry of Agriculture, in charge of agricultural and rural economic development, assumed the supervision of the quality and safety of agricultural products in the process of planting and breeding. It was not only an administration management department of agriculture, but it also supervised quality and safety of primary agricultural products. The Ministry of Agriculture was responsible for both the management of agricultural production process and the supervision of quality and safety. In order to enhance the quality and safety of agricultural products, there were three main reforms: the separation of government from enterprises, the distinction between inspection and supervision, and the integration of law enforcement teams. The so‐called agricultural comprehensive law enforcement meant that specific law enforcement agencies commissioned by the Ministry of Agriculture are centralized full‐time management departments.

The State Administration for Industry and Commerce (SAIC) was responsible for the circulation of food safety, which was the sole one, maintaining the market order to the country‐side, and was the only comprehensive law enforcement department of market access, trade, competition, and exit (FSL, 2009). The SAIC’s responsibilities included formulating specific supervision and inspection measures, implementing supervision and inspection of food quality, setting up market access standards, and investigating and punishing businesses causing major emergencies and accidents.

In 2011, China Central Television (CCTV) reported that pig farms in Mengzhou, Henan Province, used the prohibited animal drug clenbuterol to breed pigs which were sold to Shuanghui Food Co., Ltd., the largest in China and the world's leading supplier of meat (Shao & Cai, 2016). While the clenbuterol incident of Shuanghui Company occurred, the General Administration of Quality Supervision, Inspection and Quarantine (AQSIQ) was responsible for process management of food production (Jen & Chen, 2017). At the same time, China Food and Drug Administration (CFDA) assumed several responsibilities of policy and planning formulation, comprehensive coordination, major accident handling, and major information release. CFDA was established for the production, transportation, storage, distribution, and food supervision. SAIC was no longer responsible for the regulation of circulation, and AQSIQ was only responsible for the supervision of food‐related products and the supervision of import and export of food.

### 2.5 Period Five: 2015 to date

In 2015, the revised Chinese Food Safety Law promoted social cogovernance and industry self‐discipline, and strengthened the role of social groups including the food industry association, consumer federation, and media (FSL, 2015). The food industry association was required to improve industry standards and setup procedures for prosecution, provide food safety information technology and other services, guide and encourage food safety specifications of production and operations, promote the credit management of the industry, and publicize and communicate food safety knowledge. Section 4 of this article discusses the experiences obtained in Shenzhen as a typical example of promotion of social cogovernance and industry self‐discipline, involving social groups, and publicizing and communication of food safety knowledge. With the enactment of the 2015 FSL, China developed and reinforced various regulatory tools. However, there are areas of the law and regulation that need further work, such as effective coordination among government agencies, a focus on appropriate risk communication, facilitating social governance and responsibility, nurturing a food safety culture from bottom‐up, and assisting farmers at the primary level (Roberts & Lin, 2016).

CFDA established the information inquiry platform for food safety supervision and inspection, which covers sampling information, published by the general administration in 2015, and is updated in real time based on sampling inspection. To aid the functioning of CFDA, several platforms have been established. A typical example is the national food safety traceability platform, which is available for producer, government, and population (http://www.chinatrace.org). This platform was established by GS1 China, an affiliate of AQSIQ. The application was established as a demonstration project on food safety quality traceability.

While the Chinese government has developed more stringent regulatory measures and established the State Administration for Market Regulation (SAMR) in April 2018, the intermediary of current regulatory system may be the Achilles' heel. Local government is responsible for providing unified leadership, organization, and coordination of food safety supervision and management of the administrative regions, as well as food safety emergency responses, supervision and management mechanism, and information sharing mechanism.

## 3 CURRENT CHALLENGES AND WEAKNESSES

Food safety is a complex system, involving raw materials, production environment, food production, processing, transportation, storage technology, and equipment. The emergence of new media, especially Internet‐based social media, such as WeChat and Weibo in China, has transformed the way people perceive food safety information and has even surpassed the government media in terms of gaining the attention of consumers. In 2016, according to online public opinion, the main food events reported were alcohol (1900, 10.21%), meat and meat products (1627, 8.74%), vegetables and vegetable products (1569, 8.43%), aquatic products (1,388, 7.46%), and fruit and fruit products (1,372, 7.37%) (Hong & Wu, 2017). Based on total proportion, food safety events are concentrated in food production and processing (66.91%), followed by consumption (21.18%), circulation (6.42%), and production of primary agricultural products (5.49%). Food safety incidents caused by human factors accounted for 66.18% of the total, including illegal use of food additives (30.71%), fake and shoddy products (19.81%), and materials and products that are sold outside the general requirements for food labeling (7.6%). It was notable that the overuse of food additives was the primary factor. The main reasons were excessive use of preservatives in order to extend the shelf life of the product, illegal use of sweeteners to improve the taste of products, illegal use of colorants to modify product appearance, illegal use of sulfite bleaching treatment in the production process, and illegal use of stabilizers in order to prevent or delay normal food fading, oxidation, rancidity, turbidity, and flavor changes (Liu et al., 2018). Natural factors include pathogenic microorganisms (18.85%), pesticides and veterinary drug residues (9.29%), heavy metals (4.33%), and impurity substance (1.35%). Food safety systems comprise of individual parts that include science‐based research, culture, international trade agreement, food laws, and industry standards. These sectors operate together as a mechanism to integrate food safety frameworks. Any constraint to this system such as resources, technical skills, government support, and food laws may hinder the efficient functioning of food safety. Therefore, food safety regulation may be considered from three dimensions: stakeholders, technology, and management.

### 3.1 Food safety stakeholders

At present, the Engel coefficient, which measures the proportion of income spent on food, in developed countries is generally below 15%, and the corresponding food safety scores are more than 80 points. Thus, there is a phased relationship between food safety and the level of economic development. China's Engel coefficient has improved from 57.5% (urban) and 67.7% (rural) in 1978 to 36.3% (urban) and 40.4% (rural) in 2011 (Ma, 2012). The current Engel coefficient of Chinese residents was 39.4% (NDRC, 2018), which means China is still in the risk‐prone period of food safety. In addition to fulfilling basic survival needs, food is widely perceived as an ordinary commercial commodity for making profit, and thus, several incidents of illegal activities have occurred, in pursuit of increased margins, by food producers and manufacturers that have jeopardized the public's trust in food safety (Lam, Remais, Fung, Xu, & Sun, 2013).

Water scarcity, overapplication of pesticide, and chemical pollutants are considered to be the most important environmental factors impacting on food safety in China (Lu et al., 2015; Zhang, Zhong, Liu, & Ouyang, 2015). There are more than 200 million farmers engaged in cultivation who use about half a million tons of pesticides, 60 million tons of fertilizers, and 2.5 million tons of agricultural plastic films every year. Extensive agricultural production in China leads to chemical pollution (Lu et al., 2015), which is the biggest risk of current food safety. At the same time, about 20% of cultivated land in China exceeds the pollution standard of heavy metal or organic substances, and about 60% of groundwater monitoring points show poor to extremely poor (Hu, 2016).

Food industry, accounting for >10% of the total industrial output value of China, has become the largest industry and important pillar of the national economy. However, compared to the food supply system with centralized production and orderly circulation in developed countries, the food industrial structure in China is still multiple, fragmented with about 10% enterprises certificated for HACCP (Hu, 2016). Another major factor in Chinese food safety system is food adulteration or food fraud which seriously affects consumer confidence in food supply. In 2008, a fatal incident involved melamine contamination of baby milk powder which caused six deaths and several thousands of illnesses. Another serious food safety incident involved the illegal use of carcinogenic red dye Sudan Red I in chicken products in 2005. The illegal use of clenbuterol hydrochloride in animal feed caused several foodborne illnesses in 2009 (Everstine, Spink, & Kennedy, 2013; Jia & Jukes, 2013; Yan, 2012).

Food adulteration or fraud, such as the illegal addition of none approved food substances and forbidden drugs to food products, has long been a key regulatory content by Chinese government. The list of nonedible substances published by the National Health and Family Planning Commission includes several dozen kinds of melamine, formaldehyde, Sudan Red, and malachite green. However, law enforcement resources have become major constraints to the management of food control systems. After 2013, the per capita regulatory task of China Food and Drug Administration increased significantly. There are nearly 300,000 employees supervising about 12 million food production and operation enterprises, compared to the U.S. Food and Drug Administration with about 15,000 employees to about 50 thousand companies (Hu, 2016).

Several food scandals including adulteration, microbial contamination, excessive pesticide residues, and additives have continually raised consumers’ anxieties about food safety. Scattered farmers and small firms constitute the main body of food production. Due to the urgent need to improve their living standards or their earnings, there are different levels of nonstandard production and operation of food products. Moreover, due to the lack of integrity and morality, the economic penalties, and legal sanctions, under the increasingly severe pollution conditions, it is inevitable to experience frequent occurrence of food safety incidents. Another prominent incident involving the presence of cadmium in rice in 2013 was reported by the Guangzhou Food and Drug Administration. The incident on cadmium rice induced extreme panic among southern residents (Zong, 2013).

### 3.2 Food safety technical regulations

Since early 2009 to 2017, the Ministry of Health working with other ministries and relevant industry associations developed > 4,800 food standards. During the same period, these organizations integrated > 1,200 standards, including contaminants, mycotoxins, pesticides, food additives, nutritional supplements, prepackaged food labels and nutrition labels, general basic standards, dairy products, wine, food products, hygiene standards, test methods, and other special standards. Nearly 12,000 basic indicators of food raw materials and processed food were produced to limit the main health effects of food safety hazards. A National Microbiological Monitoring Network was established in 2010 according to 2009 FSL, which covers all the 31 provinces, major municipalities, and autonomous regions in Mainland China. All the major foodborne pathogens were monitored which included *Salmonella, Cronobacter* spp., *Bacillus cereus, Staphylococcus aureus, Literia monocytogenes, Vibrio parahaemolyticus, Campylobacter jejuni, and Clostridium botulinum,* as well as hygiene indicator bacteria, such as *E. coli* and other *Enterobacteriaceae* (Wu & Chen, 2018). The identification of food safety as a national priority has driven modernization of the food safety legislative framework along with organizational change, leading up to the creation of China's National Center for Food Safety Risk Assessment (CFSA), as a key contributor to the food safety standard setting process (Zhang, Godefroy, Lyu, Sun, & Fun, 2018). Meanwhile, efforts of government and food industry are not recognized by consumers, and the credibility of the government is decreasing due to poor communication of food safety risk with the public.

Traceability provides a trace‐back system which may provide consumers with assurances throughout the supply chains. The system aims to reduce the risks of foodborne diseases. However, a compulsory national animal product traceability system has not yet been established in China due to the limitations of resources, such as sponsors, availability of technology, and data standardization (Bai et al., 2017). For instance, to build the food traceability system may be not costly, but it requires budget for consumables and efficient management of the workforce. Although the existing system implemented in Beijing can provide consumers with traceability information, most consumers do not have confidence in the enquiry system as they are unable to make informed decisions about the safety of the food (Wang, Yue, & Zhou, 2017). Thus, the system should not only realize effective traceability, but also provide information about the evaluation of quality in the production chain. This approach may provide information to strengthen the food safety management system including traceability.

### 3.3 Food safety supervision system

#### 3.3.1 Food safety regulatory agencies

**Figure 1:**
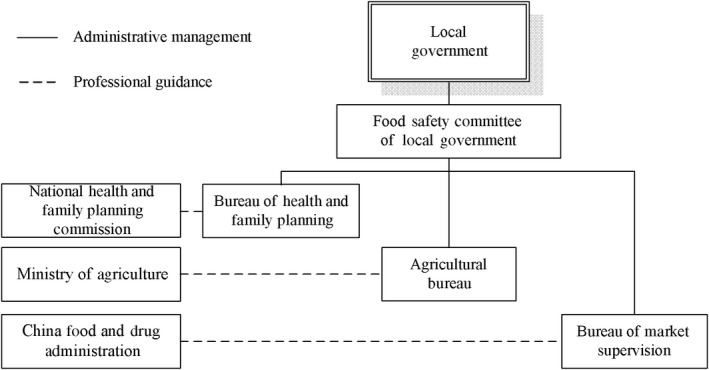
The relationship of food safety regulatory agencies in China

According to the Food Safety Law of China, Food Safety Committee of the State Council administers the overall guidance of food safety, while the National Health and Family Planning Commission is responsible for formulating and promulgating the national food safety standards. China Food and Drug Administration (CFDA) is responsible for food safety supervision from processing to the table with the exception of planting, breeding, and slaughter. Under the CFDA, slaughter is not part of processing. The Ministry of Agriculture is responsible for the supervision of the quality and safety of agricultural products during planting, breeding, and slaughter. The local government above the county level has the food safety responsibility of the region under the jurisdiction, as shown in Figure 1.

**Figure 2:**
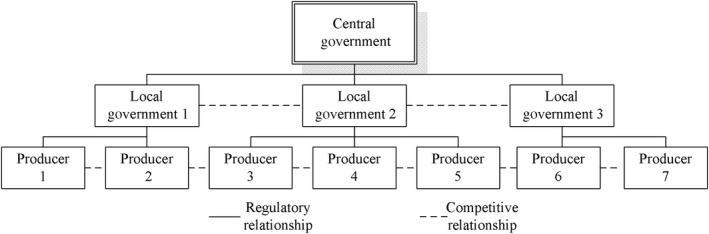
The multiple principal‐agent relationship of food safety supervision in China

The local government is responsible for food safety information. At the same time, the local government accepts the leadership of the central government and is responsible to the central government, which forms a principal‐agent relationship under asymmetric information. Meanwhile, food producers are in a position of information superiority over local governments and are regulated by the local governments. Therefore, the relationship of the central government, local governments, and food producers is multiple principal‐agent relationship. Under the standard of fiscal decentralization and political assessment, local governments are both food safety regulatory agencies and regional competitive entities, so the collusion between local governments, or different counties, and enterprises becomes a rational choice (Tirole, 1986). The multiple principal‐agent relationship of food safety supervision in China can be simplified as shown in Figure 2.

#### 3.3.2 Food safety policy

Post‐inspection increasingly reflects its limitations due to delayed reaction, expensive cost, and difficulties for effectively identifying the industry chain responsibility (Unnevehr & Jensen, 1999). HACCP is a food safety management system which focuses on prevention of potential hazards in food. The food safety system does not only prevent food safety hazards (Pierson and Corlett, 1992), but also reduces postprocessing inspections and waste thereby increasing output. HACCP has been widely adopted by many companies in countries with developed economies (Merican, 1996).

In China, the concept of HACCP was introduced in 1990, when the State Import and Export Commodity Inspection Bureau organized the Research and Application Plan of Export Food Safety Engineering. At its inception in China, HACCP was adopted by 250 food enterprises including aquatic products and other ten categories (CNCA, 2015). In 2002, AQSIQ issued regulations on hygiene registration for export food manufacturers for the first time. HACCP was compulsorily required for high‐risk products such as canned food, aquatic products, meat, fruits and vegetables, and quick frozen convenience foods. In 2003, the Ministry of Health issued the Action Plan for Food Safety, which required food production enterprises to intensively promote the implementation of the HACCP system. With the development of Food Safety Law in 2009, food production enterprises were encouraged to establish the HACCP system and strengthen ex ante control of food safety. The major problem was on the implementation and monitoring of the food safety system, since both melamine contamination formula and clenbuterol meat were produced by HACCP‐certified enterprises. The melamine‐contaminated milk incident in China was one of the most serious events considering that young children are relatively vulnerable to food contaminants. The Chinese Ministry of Health reported that 294,000 children were diagnosed with melamine‐related urinary stones, of whom 51,900 were hospitalized and at least six children died (MOH, 2008). Melamine is a nitrogen‐rich organic compound and an intermediate chemical frequently used for the manufacture of fertilizers, plastics, laminates, paints, and adhesives, and was illegally added to foods to increase their protein contents, resulting in the melamine‐contaminated milk powder scandal in China. The chemical compound harmed the health of many infants and young children which led to global attention of the fatal incident (Lancet, 2009; Li, Song, & Wen, 2019; Pei et al., 2011). Clenbuterol was used as a feed additive to promote leanness in livestock raised for their meat, and it can cause harmful effects in humans, such as inducing malignancies, chromosomal aberrations, metabolic disorders, hypokalemia, and other acute types of poisoning (Shao & Cai, 2016). In response to the continuous public demands for strengthening food safety governance, China adopted effective rules, norms, approaches, and good practices to food safety governance, including social governance. The initiatives lead the development of the 2015 Food Safety Law provisions to promote corporate social responsibility, as well as create a sustainable and effective food safety culture and behavioral change (Roberts & Lin, 2016). Besides, a report on the development of food safety regulatory systems in EU and China recommended additional measures such as training and grants to improve the capacity of the private sector to regulate China's food safety systems (Chen, Wang, & Song, 2015).

## 4 COREGULATION IN SHENZHEN

On the current situation in China, due to asymmetric information, there is a wide range of food fraud behaviors (Gong, Zhang, & Yu, 2013; Ortega, L., Wang H. H., Wu L., Olynk, & J. N., 2011; Li & Chen, 2013; Li & Shi, 2014). Meanwhile, there are regulatory captures caused by the competitive burden between local governments (Gong, Lei, & Yuan, 2015) and food producer behavioral dilemmas under severe supervision (Xie, Lai, Xiao, & Wu, 2016). Public concern about food safety is placing increasing pressure on government agencies to be more prescriptive and proactive in their regulation of the food industry. However, due to scarcity of public sector resources, concerns about the impact of regulation on competitiveness, and the current scale of the task, there is a possible model of coregulation, with public and private sectors working together to deliver safer food at lower cost (Garcia Martinez et al., 2007; Rouvière & Caswell, 2012). In 2014, the office of Food Safety Commission of the State Council created national food safety demonstration cities in four pilot provinces (Hebei, Shandong, Hubei, and Shaanxi) to promote food safety management system and encourage the improvement of national food safety governance level. In September 2015, Shenzhen was listed as one of the second batches of pilot cities by the office of Food Safety Commission of the State Council. Shenzhen occupies 1,991.64 square kilometers and its neighbor is Hong Kong. The city is located in the southern tip of the Chinese mainland and on the eastern bank of the Pearl River. By the end of 2015, there were about 11 million permanent residents in the city (China Daily, 2016). Shenzhen, the country's first special economic zone, has been a touchstone for China's reform and opening‐up policy since 1980. Its export, valued at just under 2 trillion Yuan (USD 0.23 trillion), has topped the nation's large‐ and medium‐sized cities for 26 consecutive years. Shenzhen strives to play a pivotal role in the implementation of the Belt and Road Initiative to build itself into a gateway along the 21st Century Maritime Silk Road. In this national initiative, the city can take advantage of its geographic location as well as resources in business and human resources, thereby strengthening the development of the Guangdong‐Hong Kong‐Macao Big Bay Area. Shenzhen was ranked the first in the “Thumbs up for Your City” campaign of the Food Safety Committee. The successful experiences of Shenzhen showed that once the coregulation system was formed, it needs to be supported at the onset by the regulatory authorities with finance, propaganda, and legitimacy.

### 4.1 Food safety coregulatory agencies

In order to use auditing as an inspection method rather than placing excessive emphasis on end‐product testing, the government of Shenzhen integrated resources of different food safety stakeholders to improve the overall food safety governance system. Shenzhen Institute of Standards and Technology is responsible for the development of food safety technical standards, comparing with Europe and America and other developed regions, including food traceability, propaganda and risk communication, and public opinion monitoring work.

Shenzhen Academy of Metrology & Quality Inspection has undertaken over 70% of supervision and sampling tasks. The inspectorate issues over 100 thousand food safety inspection reports throughout the year. Other organizations involved include the Shenzhen Agricultural Product Quality Safety Inspection and Testing Center and Shenzhen Institute of Drug Inspection and Testing. The sampling rate of food and agricultural products in the city increased from nearly four batches per 1,000 people in 2014 to nine batches in 2017. The qualified rate of daily supervision increased from about 95% in 2013 to about 97% in 2016 (Xie, Liu, Xiao, & Liu, 2017).

In order to boost consumer confidence, some lessons were learned from the American Consumer Union (CU) and the Stiftung Warentest (SW) (Shenzhen Online, 2017). Using the experience from these two organizations, the Shenzhen Municipal Consumer Council conducted a series of activities, such as comparative test of honey and soy sauce between Shenzhen and Hong Kong through the platform of the International Consumer Research and Testing (ICRT). This led to the establishment of the Shenzhen Food and Drug Safety Volunteer Service Group. According to the Shenzhen Food and Drug Safety Volunteer Service Management Measures (Shenzhen Online, 2019), Volunteer Shenzhen, http://v.sva.org.cn/default.aspx, is the registration platform for food and drug safety volunteers. Applicants can log on to the website and provide basic personal and necessary information on voluntary service projects, methods, and times, and the applications will be reviewed by the respective branches. After that, the Volunteer Shenzhen platform system will automatically generate a volunteer number and issue an electronic version of Shenzhen volunteer service certificate. At present, the group has about 1.7 million registered members, disseminating knowledge of food safety science, promoting food safety work, and guiding the risk control and social monitoring mechanisms in communities.

Shenzhen Retail Business Association, founded in July 1997, is a social organization comprising retail, franchise enterprises, and related individuals in Shenzhen. By 2019, there are > 500 members in the association, covering large‐ and medium‐sized outstanding enterprises such as shopping centers, department stores, supermarkets, brand chain stores, specialty stores, convenience stores, and automobile repair and maintenance chain stores. In 2015, the total sales of member companies nationwide exceeded 80 billion dollars, of which about 75% of retail sales were in or from Shenzhen. The association promotes the development of the industry and the enterprise through dozens of key projects and hundreds of events, for instance, compiling the “Food Safety Control System of Shenzhen Retail Market” in 2010, organizing “Food Safety in Shenzhen in 2011,” carrying out the self‐regulation evaluation of “Shenzhen Food Safety Standard Shop” each year, and thus has strong cohesion and appeal to the industry.

In 2012, the association and its member companies attempted to innovate the food safety social coregulation model through the assessment of food safety standard stores. Firstly, the evaluation system of food safety standard stores is mainly developed jointly by member companies. The association is responsible for the operation. The food safety regulatory authorities in Shenzhen do not participate in the daily operations, but provide policy guidance and resource supports. Secondly, member companies establish the food safety governance model through democratic consultations. The members of the jury are neither industry experts nor government agencies. Instead, the jury members are representatives of their companies for mutual learning and mutual supervision. By cycle rating system, representatives are both supervisors and the supervised. This review method not only drastically reduces the supervisory cost, but also forms a benign atmosphere for the industry to learn from each other. Finally, different from daily supervision, only qualified enterprises are publicized, while unqualified companies would obtain suggestions for rectification. In this way, the association and member enterprises successfully develop self‐organizing mode of food safety, which not only reduces the regulatory burden of government, but also increases the level of food safety management.

### 4.2 Food safety coregulatory process

The realities of food safety responsibilities have brought about a new paradigm in stakeholder relationships characterized by complex interactions between public and private modes of regulation (Fearne et al., 2004). Hence, coregulation needs to appreciate the motivations for private actors to implement enhanced food safety controls (Henson & Hooker, 2001; Hobbs et al., 2002). There are distinct differences in the established regulatory process in different countries (Garcia Martinez et al., 2007). Food safety regulation belongs to public service actively involving the government of China. Therefore, government support has an important influence on the development and operation of coregulation systems. The coregulation practice in Shenzhen has experienced three periods. First, the task of creating demonstration city exerted pressure on the transformation of food safety governance. Second, with the promotion of governance awareness and strategic transformation, all the stakeholders of food safety began to realize their duties toward a cohesive vision. Lastly, the Shenzhen Retail Business Association, supported by the government, assumed a core role of the coregulation system (Xie et al., 2017).

#### 4.2.1 Unfreezing of supervision system

The transformation of food safety governance does not only to deal with consumers' growing demand, but also alleviates the challenges encountered by regulators in daily supervision. One of the challenges is the real plight of extreme lack of regulatory resources. Although human resources, material, and financial resources invested by the regulatory authorities have increased significantly, with the acceleration of industrialization and variety of food products, they are still inadequate to meet the daily regulatory requirements. Further, the supervision department is confronted with lack of professional supervision, while the enterprises have more information about food manufacturing, circulation, catering, and other activities. Since the supervision departments became the only subject of accountability, when food safety incidents occurred, the grassroots of supervision system had to be more alert. Finally, the government of Shenzhen was committed to promoting the governance strategy from single supervision to social coregulation.

#### 4.2.2 Changing of supervision system

After changing the regulatory philosophy, the first step is to actively seek social governance subject, then the supervision can transfer part of its duties to enterprises and social organizations. Perhaps, the greatest scope for coregulation of food safety involves enforcement and monitoring rather than the establishment of regulatory standards (Garcia Martinez et al., 2007). Shenzhen Institute of Standards and Technology belongs to the Shenzhen Municipal Market and Quality Supervision and Administration Commission and is the only professional public organization on research, service, and the application of standardization. Shenzhen Retail Business Association is supported by the supervision departments to implement the assessment of food safety standard stores. The regulatory authority places emphasis on those activities from passive punishment to active prevention, by encouraging Consumer Council and volunteers to enforce the propaganda and guidance of food safety. At the same time, through use of information technology, supervisory departments focus on the supervision of high‐risk areas to achieve precision on food safety crimes.

#### 4.2.3 Refreezing of coregulation system

Since the initial stages of social coregulation system, associations and enterprises have reduced the scope for innovation in food safety governance. They rely on the government's initiative to provide policy support to assist self‐organization to acquire legitimacy. At the same time, trust mechanism is the key element of self‐organization, while government intervention objectively becomes the credit basis for attracting enterprises that are willing to participate, thereby reducing the startup cost of development. The supervision departments help the self‐organization to learn the laws and regulations of food safety, to form a benign interaction with the consumers, and construct a good institutional environment. Once the coregulation system is formed, it is supported by the regulatory authorities with finance, propaganda, and legitimacy.

**Figure 3:**
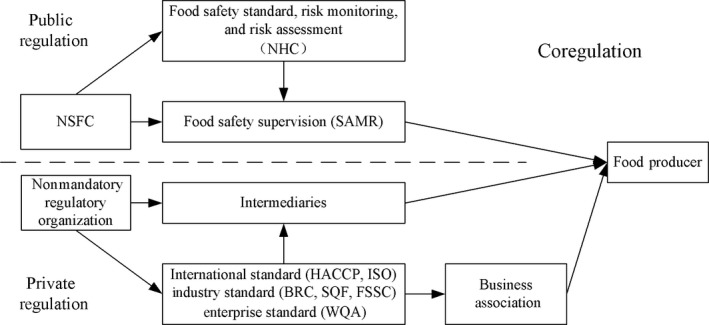
Projection of coregulation

Food safety governance in China is more than a law and policy task, as it inherently holds significant economic, political, cultural, and social implications. After a series of reforms in the past decades, China strives politically to develop and reinforce various regulatory tools, while there are areas beyond law and regulation that need to be further addressed, such as social governance, consumer trust, and behavioral change (Roberts & Lin, 2016). The experiences of Shenzhen may provide a sample, learned from other Chinese cities by shifting from a top‐down mandatory regulation to a bottom‐up coregulation, as shown in Figure 3.

## 5 CONCLUSION

Most of the food safety issues reported in China are caused by poor and ineffective food management systems. Most of the food safety accidents in China have been caused by human factors, accounted for 66.18% of the total (Hong & Wu, 2017). The review has highlighted gross abuses of food additives which have resulted in tragic losses of life. The food safety system in China is characterized by five stages comprising centralized management, multisector management, matrix management, process management, and integrated management. While the Chinese government has developed more stringent regulatory measures and established the State Administration for Market Regulation, the intermediary of current regulatory system may be still the Achilles' heel. Due to asymmetric information, lack of regulatory resources, and consumer advocacy, coregulation has been developed and is increasingly being promoted as an important instrument of regulation. The paper presents a conceptual framework of enforcement of food safety regulation for use in shifting toward coregulation from traditional approaches, based on the case study of Shenzhen. In general, the food safety governance is undergoing a shift from emphasis on punishment to advocating prevention, and supervision to promotion of cogovernance. The reform practice of food safety in Shenzhen shows that social coregulation is a feasible approach, given the coordination of government activities and other organizations including consumers. Intermediaries, such as consumer council and the third‐party quality inspection agencies, may take more important role in the future.

The concept of coregulation was developed over the last two decades and is applied to a range of economic activities. Food safety coregulation may arise, while creating new legislation or regulatory rules, by incorporating the opinions of companies, consumers, voters, nongovernmental organizations, and other stakeholders in one framework, but varying degrees of stakeholder engagement in the regulatory process and in different countries. Under the current Chinese governance scenario, especially after the national food safety strategy, government intervention has strengthened and has created opportunities to form the coregulatory system with different roles.

## CONFLICT OF INTEREST

The authors declare that we do not have any conflict of interest.

## ETHICAL STATEMENT

This study does not involve any human or animal testing. Written informed consent was obtained from all study participants.
