# Supplementary material for: Food safety governance in China: From supervision to coregulation
Source: Food Sci Nutr. 2019 Nov 20;7(12):4127–39. doi: 10.1002/fsn3.1281 (PMC6924309; doi:10.1002/fsn3.1281)
Supplement: Supplementary file 1 [file FSN3-7-4127-s001.doc]

# Abbreviations

SC: State Council of P.R.C.

AQSIQ: General Administration of Quality Supervision, Inspection and Quarantine of P.R.C.

CDC: Chinese Center for Disease Control and Prevention

CFDA: China Food and Drug Administration (was replaced by SAMR in 2018)

[CNCA](https://baike.baidu.com/item/CNCA): Certification and Accreditation Administration of the People’s Republic of China

MOA: Ministry of Agriculture

MOH: Ministry of Health (was replaced by NHFPC in 2013)

NDRC: National Development and Reform Commission

NFSC: National Food Safety Commission

NHFPC: National Health and Family Planning Commission (was replaced by NHC in 2018)

SAIC: State Administration for Industry and Commerce of P.R.C

SFDA : State Food and Drug Administration (was replaced by CFDA in 2013)

NHC: National Health Commission of the Pelple’s Republic of China

SAMR: State Administration for Market Regulation
